# Clinical Outcomes and Predictors of Poor Prognosis in Non-aneurysmal Subarachnoid Hemorrhage: A 10-Year Cohort Analysis

**DOI:** 10.7759/cureus.75596

**Published:** 2024-12-12

**Authors:** João Nogueira, Bernardo Meireles, Renato Pereira, Pedro Ribeiro, Renata Marques, Miguel Afonso Filipe, Frederica Coimbra

**Affiliations:** 1 Neurosurgery, Hospital de Braga, Braga, PRT; 2 Neurosurgery, Escola de Medicina da Universidade do Minho, Braga, PRT

**Keywords:** non-aneurysmal subarachnoid hemorrhage, non-traumatic, outcome, subarachnoid hemorrhage, wfns

## Abstract

Introduction

A large majority of spontaneous subarachnoid hemorrhages (SAH) are attributed to aneurysm rupture, though the cause remains unknown in a notable percentage of cases. Non-aneurysmal SAH (naSAH) is generally thought to follow a more benign clinical course than aneurysmal SAH (aSAH); however, similar complications may occur, and poor outcomes are still possible. Given the limited research on naSAH, this study aims to characterize these patients and correlate clinical and radiographic findings with outcomes.

Methods

A cohort of 149 patients with naSAH was selected from 2014 to 2023. Outcomes were assessed using the modified Rankin Scale (mRS), categorizing results as favorable (mRS 0-2) or unfavorable (mRS 3-6). Descriptive analysis was conducted, dividing the sample into two groups based on blood distribution on computed tomography scan: pretruncal (pnSAH) and non-pretruncal SAH (npnSAH). Associations between variables were tested, and a multivariable logistic regression was performed to identify significant predictors.

Results

The mean age was 57.54 years, with 79 males (53.0%) and 70 females (47.0%). A favorable outcome was observed in 133 patients (89.3%). Age, chronic hypertension, anticoagulant, and antiplatelet therapy were significant predictors of poor outcome (p < 0.05). A favorable World Federation of Neurosurgical Societies (WFNS) grade (I-III) was recorded in 88.6% of patients and was significantly associated with outcome (p < 0.05). Among the patients with unfavorable outcomes, 87.5% exhibited a npnSAH pattern. Rebleeding was rare, with only one case (0.7%). Acute hydrocephalus was the primary complication observed in naSAH cases (19.5%).

Conclusions

Patients with a npnSAH pattern were significantly more likely to experience unfavorable outcomes compared to those with a pnSAH pattern. In multivariate regression analysis, WFNS classification, bleeding pattern (pnSAH vs. npnSAH), and acute hydrocephalus were identified as independent predictors of poor outcomes.

## Introduction

Subarachnoid hemorrhage (SAH) is defined as the accumulation of blood in the space between the arachnoid membrane and the pia mater surrounding the brain. SAH can be classified as either traumatic SAH, the more common etiology, or nontraumatic/spontaneous [1]. Most cases of spontaneous SAH are caused by a ruptured aneurysm; however, in 10-20% of cases, no bleeding source can be identified despite repeated radiological imaging [2,3].

Spontaneous non-aneurysmal SAH (naSAH) can be further classified by blood distribution on the head computed tomography scan into pretruncal naSAH (pnSAH), which occurs in front of the brainstem, specifically anterior to the pons, and non-pretruncal naSAH (npnSAH), which presents with a more diffuse bleed pattern, with blood in the Sylvian fissure, interhemispheric fissure, or ventricles [2,4]. Non-aneurysmal SAH is generally considered to have a more benign clinical course than aneurysmal SAH (aSAH), with some studies reporting significant differences in outcomes between pnSAH and npnSAH [5].

Given the limitations in the current literature on naSAH, our study aims to comprehensively characterize a sample of naSAH patients, evaluate their clinical features, assess associated complications, and analyze the correlations between these variables and patient outcomes.

## Materials and methods

A retrospective, descriptive, analytical study was conducted on a sample of 448 patients, aged 18 years or older, diagnosed with SAH in our Neurosurgery Department from January 2014 to December 2023. Exclusion criteria were as follows: patients with aSAH, other identified causes of SAH, and no follow-up data beyond one month after the ictus.

In our department, all patients with SAH are initially evaluated with computed tomography angiography (CTA), followed by a four-vessel 3D digital subtraction angiography (DSA) to rule out intracranial sources of SAH. If the initial DSA is negative, the majority undergo a repeat DSA within 10-14 days. Additionally, magnetic resonance imaging (MRI) with a time-of-flight (TOF) sequence is performed at the first medical appointment to exclude other sources of bleeding.

All patients with SAH are treated with oral nimodipine upon admission. Specifically, in naSAH patients, screening for vasospasm with transcranial Doppler ultrasound or angiography was only conducted when there was clinical suspicion. Clinical vasospasm, also known as delayed ischemic neurologic deficit (DIND), was defined as focal neurological impairment that could not be attributed to other causes [6].

We analyzed patient characteristics, including sex, age, hypertension, smoking, alcohol consumption, antiplatelet therapy, and anticoagulation therapy. Clinical presentation at admission was assessed using the World Federation of Neurosurgical Societies (WFNS) scale, categorized into good (WFNS I-III) or poor (WFNS IV-V) presentations. Imaging characteristics were also analyzed, with bleeding patterns classified as pnSAH or npnSAH.

Statistical analysis

The data were analyzed using Statistical Product and Service Solutions (SPSS, version 29; IBM SPSS Statistics for Windows, Armonk, NY), with statistical significance set at p < 0.05 and a 95% confidence interval. Descriptive statistics included frequencies and percentages for categorical variables, as well as means and standard deviations for age. The population was examined overall and then analyzed by hemorrhage type (pnSAH vs. npnSAH) and outcome (mRS 1-2 vs. mRS 3-6).

Chi-squared tests were used to assess associations between outcomes and categorical variables. Fisher's exact test was employed when more than 20% of the cells had expected counts of less than five. The Phi coefficient was utilized to quantify effect size. Associations with age were analyzed using an independent-samples t-test, with Hedge’s g reported for effect size.

A multivariable logistic regression model was run, testing significant variables from previous tests one at a time in hierarchical order to see if they were contributing to the model and not affecting its robustness. Multicollinearity was ruled out in the final model, with variance inflation factor (VIF) < 5 and tolerance > 0.2.

## Results

After applying all the exclusion criteria, a total sample of 149 patients with naSAH was included, representing 33.3% of the original sample (448 patients). Of these, 87 (58.4%) were diagnosed with pnSAH and 62 (41.6%) with npnSAH.

Descriptive analysis of patient demographics, clinical characteristics, and outcomes

Overall patient demographics, admission status, imaging characteristics, and complications were analyzed and divided into two groups: patients with a pnSAH pattern and patients with a npnSAH pattern (Table 1). The association between these factors and outcomes was also evaluated (Table 1).

**Table 1:** Frequencies of patient history, clinical and imaging characteristics, and complications and outcomes at three months (distributed between pnSAH and npnSAH). mRS – modified Rankin Scale; No. – number of patients; npnSAH – non-pretruncal non-aneurysmal subarachnoid hemorrhage; pnSAH – pretruncal non-aneurysmal subarachnoid hemorrhage; sd – standard deviation; WFNS - World Federation of Neurosurgical Societies

Variables	Total of Patients, n (%)	pnSAH, n (%)	npnSAH, n (%)
Total No.	149 (100%)	87 (58.4%)	62 (41.6%)
Mean Age (range) ± sd	57.54 (19-92) ± 13.3	54.06 (19-87) ± 11.5	62.42 (24-91) ± 14.2
Sex			
Male	79 (53.0%)	48 (55.2%)	31(50.0%)
Female	70 (47.0%)	39 (44.8%)	31(50.0%)
Arterial Hypertension	60 (40.3%)	29 (33.3%)	31 (50.0%)
Smoking	19 (12.8%)	13 (14.9%)	6 (9.7%)
Alcohol	7 (4.7%)	3 (3.4%)	4 (6.5%)
Antiplatelet Therapy	15 (10.1%)	7 (8.0%)	8 (12.9%)
Anticoagulant Therapy	8 (5.4%)	1 (1.1%)	7 (11.3%)
WFNS scale			
I-III	132 (88.6%)	81 (93.1%)	51 (82.3%)
IV-V	17 (11.4%)	6 (6.9%)	11 (17.7%)
Seizures at admission	3 (2.0%)	2 (2.3%)	1 (1.6%)
Rebleeding	1 (0.7%)	1 (1.1%)	0 (0.0%)
Radiographic Vasospasm	8 (5.4%)	2 (2.3%)	6 (9.7%)
Clinical Vasospasm	5 (3.4%)	1 (1.1%)	4 (6.5%)
Acute Hydrocephalus	29 (19.5%)	12 (13.8%)	17 (27.4%)
Chronic Hydrocephalus	9 (6.0%)	4 (4.6%)	5 (8.1%)
OUTCOME			
Good (mRS 0-2)	133 (89.3%)	85 (97.7%)	48 (77.4%)
Poor (mRS 3-6)	16 (10.7%)	2 (2.3%)	14 (22.6%)

The mean age of the sample was 57.5 ± 13.3 years, with pnSAH patients averaging 54.06 ± 11.5 years and npnSAH patients averaging 62.42 ± 14.2 years. The sample included 79 males (53%), with a similar sex distribution across groups. Hypertension was observed in 40.3% of patients, with a higher prevalence in the npnSAH group (50%). Smoking prevalence was 12.8%, and 4.7% of patients reported alcohol consumption. The rate of patients on antiplatelet therapy was 10.1%, and 5.4% were on anticoagulants, primarily in the npnSAH group (11.3%).

WFNS grades I-III were recorded in 132 patients (88.6%), with a higher prevalence in the pnSAH group (93.1%) compared to the npnSAH group (82.3%). Poor clinical presentation, classified as WFNS grades IV-V, was more common in the npnSAH group (17.7%) than in the pnSAH group (6.9%).

Seizures at admission were rare, occurring in only three patients (2.0%). Rebleeding occurred in only one patient (0.7%). Radiographic vasospasm was observed in eight patients (5.4%), with a higher incidence in the npnSAH group (9.7%). Clinical vasospasm occurred in five patients (3.4%), with more cases in the npnSAH group (6.5%).

Acute hydrocephalus requiring a ventricular drain was present in 29 patients (19.5%), with a higher prevalence in the npnSAH group (27.4%) compared to the pnSAH group (13.8%). Chronic hydrocephalus was reported in nine patients (6.0%), with the need for permanent shunting.

Regarding the outcome of these patients, a poor outcome (mRS 3-6) was observed in 16 patients (10.7%): 14 patients in the npnSAH group, compared to only two patients in the pnSAH group.

Demographic and clinical predictors of outcome

The association between all these factors was also evaluated (Table 2).

**Table 2:** Associations between patient history, clinical and imaging characteristics, complications, and outcomes. The values in bold stand for statistically significant differences (p < 0.05). Fisher – Fisher’s exact test; g – Hedge’s g; mRS – modified Rankin Scale; No. – number of patients; npnSAH – non-pretruncal non-aneurysmal subarachnoid hemorrhage; pnSAH – pretruncal non-aneurysmal subarachnoid hemorrhage; t – Independent Sample T-Test; V – Cramer’s V; WFNS – World Federation of Neurosurgical Societies; ϕ - Phi for effect size;χ2 – Chi-square test

Variables	Good Outcome (mRS = 0-2), n (%)	Poor Outcome (mRS = 3-6,) n (%)	Statistical Test	p value	Effect Size
No. of patients	133 (89.3%)	16 (10.7%)			
Age	55.23 ± 11.8	76.75 ± 9.5	t = -7.027	<0.001	g = -1.850
Sex			χ^2^ = 0.618	0.432	ϕ = -0.64
Male	72 (54.1%)	7 (43.7%)			
Female	61 (45.9%)	9 (56.3%)			
Hypertension	45 (33.8%)	15 (93.8%)	χ^2^ = 21.315	<0.001	ϕ = 0.378
Smoking	19 (14.3%)	0 (0.0%)	Fisher = 2.620	0.225	ϕ = -0.133
Alcohol	7 (5.3%)	0 (0.0%)	Fisher = 0.884	1.000	ϕ = -0.077
Antiplatelet Therapy	9 (6.8%)	6 (37.5%)	Fisher = 14.900	0.002	ϕ = 0.316
Anticoagulant Therapy	4 (3.0%)	4 (25.0%)	Fisher = 13.596	0.005	ϕ = 0.302
WFNS scale			Fisher = 26.410	<0.001	ϕ = 0.421
I-III	124 (93.2%)	8 (50.0%)			
IV-V	9 (6.8%)	8 (50.0%)			
pnSAH vs npnSAH			χ^2^ = 15.536	<0.001	ϕ = 0.323
pnSAH	85 (63.9%)	2 (12.5%)			
npnSAH	48 (36.1%)	14 (87.5%)			
Seizures at admission	2 (1.5%)	1 (6.3%)	Fisher = 1.631	0.291	ϕ =0.105
Rebleeding	1 (0.8%)	0 (0.0%)	Fisher = 0.121	1.000	ϕ = 0.728
Radiological Vasospasm	6 (4.5%)	2 (12.5%)	Fisher = 1.794	0.206	ϕ = 0.180
Clinical Vasospasm	3 (2.3%)	2 (12.5%)	Fisher = 4.622	0.089	ϕ = 0.032
Acute Hydrocephalus	18 (13.5%)	11 (68.8%)	Fisher = 27.779	<0.001	ϕ = 0.432
Chronic Hydrocephalus	5 (3.8%)	4 (25.0%)	Fisher = 11.277	0.008	ϕ = 0.276

Age (p < 0.001, g = -1.850), hypertension (p < 0.001, ϕ = 0.378), and both antiplatelet (p = 0.002, ϕ = 0.316) and anticoagulant (p = 0.005, ϕ = 0.302) therapies were associated with poorer outcomes. The clinical presentation, measured by the WFNS score, was significantly associated with patient outcomes (p < 0.001), with a moderate-to-large effect size (ϕ = 0.421). A significant difference in outcomes between patients with different bleeding patterns (pnSAH vs. npnSAH) was also observed (p < 0.001), with an effect size of ϕ = 0.323, suggesting a moderate association.

There was a significant association between acute hydrocephalus and outcome (p < 0.001), observed in 68.8% of patients with poor outcomes. The effect size (ϕ = 0.432) indicates a moderate-to-large effect. While chronic hydrocephalus was also significantly associated with poor outcomes (p = 0.008), its effect size (ϕ = 0.276) suggests a weaker association.

There was no significant correlation between vasospasm and outcome (p = 0.206) nor was there a correlation with rebleeding, which was only observed in one case.

Multivariate analysis of key predictors for poor outcomes

A multivariate logistic regression analysis was conducted using three clinically relevant variables: bleeding pattern, WFNS score, and acute hydrocephalus (Table 3). All three variables were significant predictors of poor outcomes. Higher WFNS scores were strongly associated with unfavorable outcomes (OR = 7.511 (1.770; 31.871); p = 0.006), while acute hydrocephalus increased the likelihood of a poor outcome with an OR of 9.297 (2.427; 35.612) (p = 0.001). A higher risk of poor outcomes in patients with npnSAH was again confirmed, revealing the highest odds ratio of the three (OR = 13.178 (2.282; 76.109); p = 0.004).

**Table 3:** Multivariate logistic regression with selected significant variables. The values in bold stand for statistically significant differences (p < 0.05) and respective OR. B – regression coefficient; S.E. – standard error; OR – odds ratio; CI – confidence interval

Variables	B (S.E.)	Wald	OR (95% CI)	p value
WFNS scale	2.016 (0.737)	7.476	7.511 [1.770; 31.871]	0.006
pnSAH vs npnSAH	2.579 (0.895)	8.305	13.178 [2.282; 76.109]	0.004
Acute Hydrocephalus	2.230 (0.685)	10.590	9.297 [2.427; 35.612]	0.001

## Discussion

The percentage of naSAH was 33.3%, which is higher than the approximate range of 10-20% stated in previous studies [2,3]. The specific cause for this discrepancy remains unknown, even though all patients at this study's center underwent CTA, one (or sometimes two) DSA, and an MRI with TOF sequence during follow-up, without revealing a possible cause for the SAH.

Our study identified hypertension as a significant predictor of poor outcomes in naSAH. This finding challenges other recent literature that found no such association in naSAH, despite hypertension being a well-established factor for adverse outcomes in aSAH [5]. The mechanisms may involve cerebral ischemia and dysregulation of cerebral blood flow [7]. Antiplatelet and anticoagulant therapies were also identified as factors for poorer outcomes. Though data on these drugs in naSAH are limited, there is an association with higher blood volume and rebleeding rates [8]. One study found that poor outcomes were more common in patients on antiplatelet therapy, though this difference was not significant at 12 months follow-up [9]. These medications may reflect the presence of underlying comorbidities, which may contribute to poorer outcomes. Since our study assessed outcomes only at three months, further research is needed to determine whether these associations persist over a longer term. Future studies can investigate other cardiovascular factors, such as atrial fibrillation, diabetes, or petechial hemorrhage, as has been done in other cerebrovascular diseases [10,11].

The WFNS scale was a significant predictor of poor outcomes, consistent with findings from previous studies [5,8,12,13]. However, there are concerns about the scale’s predictive power. Recent studies have shown that several patients with high WFNS scores at admission can still achieve good outcomes [14]. Similarly, in the present study, more than half of the patients (52.9%) with WFNS grades 4-5 at admission experienced favorable recoveries.

Seizure incidence at admission was very low, with only three cases observed (2.0%). This could be attributed to the difficulty in identifying seizures that occur at the onset of symptoms, which often go unreported or undetected [15].

The proportion of pnSAH (58.4%) and npnSAH (41.6%) cases in this study is comparable to existing literature [3,5]. There was a clear difference between pnSAH and npnSAH bleeding patterns, with npnSAH patients not only being older, more likely to have a history of hypertension and anticoagulation therapy, but also having higher WFNS grades and higher rates of acute hydrocephalus. These patients were also more likely to have a poor outcome (87.5% of poor outcome patients had a npnSAH pattern), which was confirmed by the multivariate regression and aligns with findings from previous studies [2,16]. These studies suggest that npnSAH represents an intermediate pattern between pnSAH and aSAH in terms of outcome. A poor outcome was reported in 2.3% of pnSAH patients and 22.6% in npnSAH patients, compared to an incidence of 42.3% in aSAH found in a previous study [17].

Rebleeding was reported in only one case (0.7%), which aligns with previously reported rates in the literature, being extremely rare in naSAH, ranging within 0-4% [18].

Hydrocephalus was a common complication, occurring in 19.5% of patients, with 6.0% progressing to chronic hydrocephalus, requiring shunting. These rates are consistent with prior studies that have reported acute hydrocephalus in 11-30% of naSAH cases and chronic hydrocephalus in 3.7-17% of patients [2,3,5,8,18]. The differences in its incidence between pnSAH (13.8%) and npnSAH patients (27.4%) were notable, confirming data from previous studies and reinforcing the importance of pnSAH vs npnSAH classification [3,17]. Acute hydrocephalus was a significant predictor of poor outcomes, as also seen in previous studies [2,5,8,18]. This finding was confirmed in the multivariate analysis, stating that having acute hydrocephalus increases the probability of a poorer outcome by approximately nine times. The underlying mechanisms likely involve impaired cerebrospinal fluid (CSF) circulation and increased intracranial pressure, which reduce cerebral perfusion and cause ischemia and brain tissue compression, leading to neurological deficits, including cognitive impairment, motor dysfunction, poorer functional recovery, and increased mortality-justifying the poorer outcomes [19].

Vasospasm rates were lower in this study compared to others, with 5.4% for radiographic vasospasm and 3.4% for clinical vasospasm. Previous studies have reported higher rates, ranging between 10% and 16% for radiographic and 6% and 10% for clinical vasospasm [2,5,18]. This discrepancy, particularly regarding radiographic vasospasm, may be attributed to differences in the use of CTA or DSA between this study’s center and others. At our center, there was no established protocol for periodic CTA or DSA; these imaging modalities were performed only when clinically indicated, which may have led to the underdiagnosis of radiographic vasospasm.

The multivariate logistic regression was based on the clinical relevance and statistical significance of each variable. With a sample of 149 patients and the relatively benign nature of this condition, the study had only 16 events of poor outcomes, which typically limits the model to two variables, following the rule of 10 events per variable. However, this general rule is flexible [20]. Consequently, by using hierarchical variable entry, along with adjustments for multicollinearity and control of outliers, the model was extended to include three variables, enhancing the study’s clinical relevance while maintaining its robustness. Nevertheless, some odds ratios (ORs) and their confidence intervals were higher than typically expected [20]. Therefore, their predictive value should be interpreted with caution, considering these limitations. Despite this, the multivariate analysis results clearly demonstrate the importance of these three factors in determining outcomes for naSAH patients. The bleeding pattern (pnSAH vs npnSAH) showed the highest predictive value, increasing the risk of having an unfavorable outcome by 13 times, reinforcing this variable as a crucial factor in managing these patients. Furthermore, WFNS and acute hydrocephalus are decisive factors to consider during both acute management and follow-up, as they remained independently significant predictors for outcomes.

Limitations

This study has several limitations. Firstly, being retrospective in nature, it is susceptible to variability and potential bias in the classification of clinical scales, as well as incomplete data due to missing documentation in medical records. Additionally, the relatively small sample size, derived from a single center, limits the generalizability of the findings. Larger, multicenter studies would help strengthen the validity of these results and improve their broader applicability. Another potential limitation is the possibility that patients with mild symptoms of naSAH may not have sought medical attention, which could lead to an overestimation of complications, such as acute hydrocephalus and poor outcomes. Finally, the study assessed outcomes only at three months post-ictus using the mRS, which may not fully capture long-term recovery. This limitation is compounded by patient loss to follow-up, which also contributed to a reduced final sample size.

## Conclusions

A good outcome was achieved in most patients, reinforcing the notion of a relatively benign course compared to aSAH. However, there were clear differences in outcomes between pnSAH and npnSAH, with npnSAH accounting for the majority of poor outcomes. This highlights the importance of this classification and underscores the need for increased vigilance when monitoring npnSAH patients.

Acute hydrocephalus emerged as the most common complication. Age, hypertension, chronic use of antiplatelet or anticoagulant therapy, and hydrocephalus were all significantly associated with poor outcomes. A multivariate analysis identified three variables- bleeding pattern (pnSAH vs. npnSAH), acute hydrocephalus, and WFNS score - independently significant predictors of poor outcome. These factors should be considered when monitoring patients with naSAH.
